# “*You are the first person to ask me how I’m doing sexually*”: sexual and reproductive health needs and sexual behaviours among migrant people in transit through Panama

**DOI:** 10.3389/frph.2023.1157622

**Published:** 2023-07-10

**Authors:** Sofya Panchenko, Philippe Mayaud, Sebastian Baranyi Nicholls, Carolina López González, Khatherine Michelle Ordáz, Madeline Baird, Amanda Gabster

**Affiliations:** ^1^ Faculty of Infectious & Tropical Diseases, London School of Hygiene and Tropical Medicine, London, United Kingdom; ^2^ Instituto Conmemorativo Gorgas de Estudios de la Salud, Panama City, Panama; ^3^ Faculty of Medicine, University of Ottawa, Ottawa, ON, Canada; ^4^ Faculty of Medicine, Universidad Interamericana de Panamá, Panama City, Panama; ^5^ Faculty of Latin American and Social Studies, FLACSO, Buenos Aires, Argentina; ^6^ Faculty of Anthropology, Univeristy of Connecticut, Storrs, CT, United States; ^7^ National Research System of Panamá (SNI), Panamá, Panamá; ^8^ Center of Population Sciences for Health Equity, College of Nursing, Florida State University, Tallahassee, FL, United States

**Keywords:** migrant, migrant healthcare, sexual and reproductive health (SRH), sexually transmitted infections (STI), HIV, sexual violence, Darien, Panama

## Abstract

**Background:**

Unprecedented numbers of migrant people transiting through the Darién Gap at the Panama-Colombia border were recorded in 2021 and 2022. Data on sexual and reproductive health (SRH) needs and service provision among migrant people in transit is generally extremely sparse. This study aimed to collect personal accounts of sexual behaviours and SRH needs and access to services among migrant people in transit through Panama.

**Methods:**

We conducted a rapid-assessment qualitative study using semi-structured interviews during June-July 2022. Participants were migrant people in transit at three locations across Panama: (i) at the Migrant Reception Station (MRS) in Darién province at the Panama-Colombia border, (ii) in the city of David near the Costa Rica-Panama border, and (iii) at the Costa Rica-Panama border. Migrant peoples (>18 years) were invited to participate using purposive sampling.

**Results:**

Overall, 26 adult migrant people (16 men, 10 women) across the three sites participated in the study. We identified three overarching themes from the interviews: (1) increased need for SRH service provision, (2) experiences of sex, relationships, and transactional sex, and (3) vulnerability to exploitation and sexual violence. All accounts reported that no formal SRH care was present during the journey through the Gap and described as inconsistent at the MRS in Darién. Provision of gynaecological or genital examinations, laboratory testing for urinary tract or STI, and prenatal care were mentioned to be the most pressing needs. Participants reported a change in their sexual behaviour while travelling, whether a decline in sexual libido or preference towards short-term partners. Most female participants recounted constantly fearing sexual violence during the journey through the Gap and several respondents reported witnessing incidents of sexual and other forms of violence.

**Conclusion:**

There are significant unmet needs regarding SRH care during the journey of migrant people transiting through the Darién Gap, at the MRS in the Darién province, and across Panama. Provision of antenatal care, rapid testing for HIV/STI, condom distribution, and care for victims of sexual violence would significantly reduce adverse SRH outcomes and improve the well-being of migrant people, even when in transit.

## Introduction

Achieving a state of physical, emotional, mental, and social well-being in relation to sexuality and reproduction relies on the realisation of sexual and reproductive rights, based on the human rights of an individual. Thus, the definition of sexual and reproductive health has been expanded at the 1994 International Conference on Population and Development (ICPD) to address the full range of people's needs and the services required for the maintenance of sexual and reproductive health (1, 2). A comprehensive interpretation of sexual and reproductive health and rights (SRHR) includes access to sex education, safe birth control methods, abortion services, HIV/STI testing and treatment services, and appropriate pregnancy and childbirth care (3).

There are major gaps in the literature on SRHR of migrant people—most of the available information pertains to specific SRHR barriers faced by migrant people after their settlement in the destination country, while the needs and experiences of those in transit remain largely undocumented. Lack of available research may be explained by the difficulty of data collection due to the temporary nature of transit, requirement for international cooperation and research funding across multiple countries, and unreliable nature of quantitative data related to migrant people's interceptions at borders (4, 5).

The term “migrant in transit” has no universal definition and does not refer to a legal category of individual; “transit migration” is commonly viewed as a process rather than a migration status, an umbrella term for various migrant categories that includes both irregular and regular migration and that may lead to vastly different migration outcomes (4, 6). Despite the existence of international human rights instruments guaranteeing the right to SRH for migrant and refugee populations, there are health disparities affecting migrant people in transit due to inadequate access to healthcare services at border sites, in transit and after settlement; the health needs of migrant people often grow during transit due to exceedingly tough physical conditions of the migratory process, the erosion of social and financial support mechanisms, and the power imbalance between human traffickers and migrant people (7, 8).

A particular scenario has developed in Panama, where border authorities receive migrant people in transit emerging from the Darién Gap—a 66-mile region along the border between Panama and Colombia that serves as a recently established migration route for those travelling North to Mexico, United States, and Canada (9, 10). According to the National Migration Service of Panama, less than 11,000 annual crossings were recorded from 2010 to 2020. By contrast, from January to October 2022, more than 156,000 people had traversed the Gap, with the overwhelming majority travelling from Venezuela (11, 12). The movement of people across the border is facilitated by human traffickers (known among migrant people as “guides” or “coyotes”) who transport those who wish to travel from Colombia by boat and through the Gap by foot en route to North America (9, 11, 13). The ubiquity of criminal activity within the Gap has been described by the Panamanian government, non-governmental organisations (NGOs), and the local media over the last twenty years (14–16). Government reports highlight trafficking routes throughout the region, pointing to the existence of “clientelist social relationships” between traffickers and the local residents (14).

Despite the large number of migrant people passing through Panama, access to relevant preventative and therapeutic services is extremely limited. Pre-existing SRH conditions may be exacerbated during the journey through the Gap due to delays in accessing care, malnutrition, dehydration, poor hygiene, and pervasive sexual violence (8). Timely detection and management of sexually transmitted infections (STIs), including HIV, and other genital infections can prevent related morbidity and mortality among, and transmission from, migrants travelling through Central America. Recent studies on transit migration within Latin America and the European Union (EU) have noted that improving access to primary health care, including STI and HIV testing, among migrant people in transit can significantly reduce future health consequences of untreated conditions, as well as provide direct and indirect economic advantages for the host countries (17, 18). HIV/STI transmission can occur partly due to high risk of sexual violence and abuse: 180 cases of rape within the Gap were reported to Doctors Without Borders during the period May-September 2021 (8, 16).

The risks of sexual assault and other forms of gender-based violence (GBV) are known to increase when unsafe or irregular migration routes are used; while GBV can be experienced by members of any gender, it disproportionately affects women, girls, and those of diverse gender identity or diverse sexual orientation (19, 20). Data on migration to and within the EU has highlighted that legal provisions on migrant SRHR often neglect to consider the extent of sexual violence experienced by highly vulnerable migrant sub-groups, such as adolescent girls and sex workers, in periods of protracted migration; this means that migrant people vulnerable to sexual violence often face significant legal obstacles in realising their rights to SRH services, which is exacerbated by inadequate personal resources and administrative barriers (21–23).

Both the characterisation of migrant people's sexual and reproductive health and an increased understanding of their needs align with the goals of Panama's Ministry of Health (MINSA) and the Pan American Health Organization (PAHO) in offering better healthcare and support to people in transit through Panama. Gabster et al’s team conducted a rapid epidemiological assessment of SRH needs and access among migrant people in transit through Panama, the results of which indicated a total lack of access to SRH care (8). This follow-up qualitative research sought to expand on the narrative experience of the migrant people in transit both prior to and during the journey through the Darién Gap and Panama.

The aims of this study were to obtain accounts of sexual behaviours, SRH needs and access to relevant services experienced by the population of migrant people in transit, both prior to and during the journey through Darién Gap, and throughout Panama. We focused on describing the individual, social, and institutional factors that may act as barriers to health-seeking behaviour and influence migrant people's SRH outcomes.

## Methods

We conducted a cross-sectional rapid-assessment qualitative study using semi-structured interviews at three different locations in Panama, with a sample of adult (≥18 years) migrant people in transit across the country.

### Research setting

The study was conducted at three locations on the migrants' journey across Panama: (i) at an MRS in Darién province on the southern Panama border with Colombia; (ii) at the main bus terminal in David, Chiriquí province, near the Costa Rica border; (iii) and at the Panama-Costa Rica border, Chiriquí province. To facilitate their travels onwards, migrant people are then transported to David and the Panama-Costa Rica border following their stay in the Darién MRS. The settings were chosen due to being at opposite sides of the country, which allowed the co-authors to see if migrant people's experiences with SRH and behaviours were different after leaving the Gap but before leaving Panama.

While at the time of the study, most migrant people travelled via the same route from Darién MRS to the Panama-Costa Rica border, all three locations were utilised due to the large density of migrant people residing at and passing through the MRS, creating a more suitable environment for purposive sampling.

There is no access to healthcare within the Darién Gap due to a complete lack of infrastructure. At the Darién MRS, the only access to a health professional is provided through visits from NGOs such as the Panamanian Red Cross and Doctors Without Borders (MSF). Two health centres in the communities of Metetí and Santa Fe, Darién province, can provide health services to migrant people in transit in case of medical emergencies. However, these care visits require authorised transport by the National Migration Service and the National Border Service of Panama (SENAFRONT) (13).

### Study population and sampling

Participants were selected using non-randomised, purposive sampling. Recruitment was undertaken based on participants' ability to communicate experiences in an expressive and reflective manner, and their availability and willingness to participate.

Purposive sampling was used to include a representative sample of men and women who were interested in speaking about their SRH experiences. Quota sampling was used to produce an approximate 60/40 percent ratio of male to female participants to represent the total number of migrant people in transit; the ratio was based on the 2021 data provided by the National Migration Service of Panama (24). Additionally, potential participants must have been ≥18 years, and to speak and read at least basic English, Portuguese, French or Russian (in order to read the consent form offered). Even if they were willing to participate in the study, individuals were not chosen if they had a planned exit from Panama within 8 h after concluding the interview in case the study team needed to refer the individual to social services.

Sampling took place in communal areas of the MRS, around the bus station and in the streets of the Panama-Costa Rica border. Co-authors approached potential participants and asked if they would be willing to hear about a study in a private location, out of earshot of others. The purpose of the study was verbally described to potential participants; those eligible and willing to participate were invited to sign a written informed consent. The interviews were undertaken in a location chosen by the participant for its privacy.

Languages used in the interviews were selected based on prior observation of these native language speakers in transit through Panama. There was no *a priori* sample size determination apart from aiming to obtain data saturation with regard to the themes and objectives of the study.

### Data collection

A semi-structured interview guide was designed to capture diverse personal experiences regarding (i) participants' journey to and through Panama, (ii) their romantic and sexual relationships, (iii) prevention, testing, and care needs related to HIV and STIs, (iv) reproductive healthcare experience, needs and access, (v) and prevention and reporting needs of experiences of violence.

Data collection was performed over 7 days, from June 30-July 7, 2022. Semi-structured interviews were conducted by trained field workers and lasted between 30 and 60 min. Participant pseudonyms and details are shown in Table 1. Interviews were recorded digitally, transcribed verbatim, and independently translated into English. Back translation was performed for a randomly selected 10% sample of interviews recorded in Spanish and French languages to ensure translation quality.

**Table 1:** Characteristics of each participant.

Pseudonym	Sex	Age	Location of interview	Participant country of origin
Osei	Male	32	Darién MRS^a^	Ghana
Luis	Male	22	Darién MRS	Venezuela
Emmanuel	Male	50	Darién MRS	Haiti
Joseph	Male	18	Darién MRS	Congo
Mauricio	Male	26	Darién MRS	Angola
Ana	Female	23	Darién MRS	Venezuela
Hector	Male	42	Darién MRS	Cuba
Isabel	Female	18	Darién MRS	Venezuela
Gabriel	Male	26	Darién MRS	Venezuela
Beatriz	Female	40	Darién MRS	Venezuela
Maria	Female	32	Darién MRS	Venezuela
Diana	Female	22	Darién MRS	Venezuela
Gloria	Female	24	Darién MRS	Venezuela
Sonia	Female	18	Darién MRS	Venezuela
Francisco	Male	26	David bus terminal	Venezuela
Jesus	Male	37	David bus terminal	Venezuela
Simon	Male	29	David bus terminal	Colombia
Carolina	Female	29	David bus terminal	Venezuela
Robert	Male	32	David bus terminal	Venezuela
Jorge	Male	28	David bus terminal	Venezuela
Rafael	Male	44	David bus terminal	Venezuela
Angelica	Female	30	David bus terminal	Venezuela
Jaime	Male	29	David bus terminal	Venezuela
Teresa	Female	20	Costa Rica-Panama border	Venezuela
Alvaro	Male	24	Costa Rica-Panama border	Venezuela
Victor	Male	36	Costa Rica-Panama border	Venezuela

^a^ MRS, Migrant Reception Station.

### Analysis

Interviews were analysed using a mixture of deductive and inductive approaches to thematic analysis developed by Braun and Clarke (25). Initial codes from the interview transcripts were generated using NVivo 12 (QSR International Pty Ltd. 2019). Transcripts were coded based on both the established *a priori* determined themes and any emerging ones, with the coding frame being constantly revisited to refine emerging themes.

### Ethics

Local approval was granted by the Research Bioethics Committee of the Gorgas Memorial Institute for Health Studies, Panama (ref: N221/CBI/ICGES/22). The study was also approved by the London School of Hygiene and Tropical Medicine Ethics Committee (ref: 27587). All participants signed written informed consent forms in a private location and were interviewed in a private location by a single interviewer, with a translator present where appropriate. Participants were told that if they wished to discuss any physical violence or sexual assault committed against them on Panamanian territory, the research team would help them report it to the relevant authorities. Participants who reported the need for healthcare or social services during the interview were linked by an interviewer to an NGO National Ministry that was providing relevant care.

## Results

### Participant characteristics

Overall, 26 adults aged >18 years (16 men and 10 women) participated in this study. Their sociodemographic characteristics are summarised in Table 2. Most participants (*n* = 20) originated from Venezuela. Median age of all participants was 28.5 years, ranging between 18 and 50 years old; 23 interviews were conducted in Spanish, 2 in French, and 1 in English. 14 interviews were conducted at the Darién MRS, 9 in the city of David, and 3 at the Costa Rican border.

**Table 2:** Sociodemographic characteristics of the participants.

	Women (*n* = 10)	Men (*n* = 16)
**Age group (years)**		
18–24	6	3
25–29	1	6
30–34	2	2
35–39	–	2
40–44	1	2
45–50	–	1
**Country of origin**		
South America		
Venezuela	10	10
Colombia	–	1
Caribbean		
Haiti	–	1
Cuba	–	1
Africa		
Ghana	–	1
Congo	–	1
Angola	–	1
**Languages spoken**		
Spanish	10	13
Portuguese	1	5
French	–	2
English	–	1
Other^a^	–	3
**Relationship status**		
Single	4	8
In relationship	6	5
Married	–	3
Divorced	–	–
Widowed	–	–
**Highest education level^b^**		
Primary school only	–	–
Some secondary school	2	3
Secondary school diploma	3	4
Some university	3	4
Bachelor's degree	2	2
Master's degree/PhD	–	1

^a^ Other language category included Creole, Lingala, and Hausa languages spoken by some participants in addition to either Spanish, Portuguese, English and/or French.

^b^ Highest education level for two participants, Joseph and Jaime, could not be recovered.

Our analysis identified three overarching themes: (1) increased need for SRH service provision, (2) experiences of sex, relationships, and transactional sex, and (3) vulnerability to exploitation and sexual violence.

#### Increased need for sexual and reproductive health (SRH) service provision

NGOs such as MSF and the Panamanian Red Cross provide general health services, including basic SRH care at the Darién MRS during day hours. Five participants (1 man, 4 women) said that they were suffering from a possible urinary tract or reproductive tract infection (UTI/RTI) at the time of interview, which they believed was acquired due to poor hygiene conditions while crossing the forest, but could not receive adequate care at the NGO stations that had been set up. Gynaecological examinations in particular were emphasised as an urgent need for migrant women as symptoms of UTIs/RTIs upon arrival to the MRS were said to be commonplace:

*“We were all sitting on the floor [of the boat] and there were a lot of children. And many women, and they all urinated there […] And since we were all sitting like that, we felt that all of it got into our private parts […] When we urinated, it was horrible, it burned.”* Isabel, 18

SRH service provision at the Panamanian MRS and throughout the route across Panama was highlighted as a neglected issue among the migrant peoples—being in an environment where sexual and reproductive care is consistently deprioritised in favour of issues that are deemed more immediate can lead to long-term consequences to both physical and mental health:

*“You’re the first person to ask me how I feel sexually [how my sexual health is], how everything is functioning for me. The first. And I’ve been to all the medical centres [throughout Panama], and this is the first time that anyone asked me”* Beatriz, 40

When asked about where the SRH services should be provided many agreed that the Darién MRS and the city of David would be the best locations since most, if not all, migrant people in transit pass through them.

##### HIV/STI awareness

Being equipped with information about prevention of HIV/STI transmission and maintaining personal hygiene was considered by most as a crucial component of sexual health maintenance. Sixteen participants identified HIV as either one of, or the only, sexually transmitted infection. Human papillomavirus (HPV), syphilis, and gonorrhoea were also commonly named. Most female participants felt that it was very important for them to know their health status regarding HIV and STIs for their own benefit and to protect others while in transit. Some, however, did not seem to worry, especially those in exclusive sexual relationships, as the way to reduce the risk of HIV/STIs was commonly believed to be *“taking care of yourself”,* being *“selective with all [sexual] partners*”, and *“using protection”*. Some participants connected their lack of worry to the fact that they *“didn't notice anything unusual on [their] body”* (Ana, 23), i.e., being asymptomatic.

There appeared to be an increased need for health promotion surrounding HIV/STIs, which was highlighted by the use of stigmatising language among some participants, as well as general misinformation. A few interviewees demonstrated erroneous knowledge of HIV/STI transmission by saying, for instance, that HIV can be transmitted by being *“in contact with the rim [of a glass/container] that has this disease”* (Mauricio, 26) or *“through saliva”* (Gabriel, 26). In one case, language that implied personal judgement about casual sexual relationships seemed to be linked to HIV stigma:

*“That gay guy was giving us water from his pot where he drinks the water from and sticks his mouth in, and how many things did he do to the other one [sexual partner] there?”* Beatriz, 40

On the other hand, those who had a personal experience with an STI (e.g., herpes) or were friends with people living with HIV could correctly describe the routes of HIV/STI transmission when prompted to, and noted that they *“don't discriminate against people who have this type of illness”* and *“want to learn more about these diseases”* (Jesus, 37).

##### HIV prevention and contraception

Many participants used the expression *“to take care of [one]self”* in relation to using birth control methods, preventing HIV/STI acquisition, and maintaining personal hygiene. Both male and female participants were able to name a variety of contraception methods that exist, such as contraceptive injections, implant, intrauterine device (often referred to as “T” due to its characteristic shape), the pill, and spermicides, either from using it themselves, from their friends, or their partners. When asked to discuss their reason for using hormonal contraception at the time of the interview, female participants said that it allows them to gain more control of their reproductive health by making a conscious choice to not become pregnant during or shortly after their journey [e.g., to*“enjoy my life in the US”* (Diana, 22)].

All female participants recognised the need to bring contraception with them if they wished to be protected from pregnancy during their journey due to complete lack of SRH services within the Darién. Some emphasised the need for birth control provision at Darién MRS and other health services while in transit, specifically for sexually active adolescent girls:

*“There are also many children who come here, who are 16, 14, who are not yet young ladies, but they get with someone […] and they are not taken care of either.”* Maria, 32

All respondents agreed that it was not possible to obtain condoms anywhere during the journey through the Darién Gap—many felt it was important for condoms to be provided at the MRS on Panamanian territory and at the camps within the forest, saying that *“[giving out condoms] should be a benefit to any camp”* (Carolina, 29), both for current and future use. An overwhelming number of testimonies recognised the lack of HIV/STI prevention and care services throughout the journey and the essential nature of them, as *“it never hurts to offer health [services]”* (Alvaro, 24).

One participant also gave an account of SENAFRONT personnel confiscating condoms upon arrival at the Darién MRS, making it difficult to practice safer sex:

*“A girl who was right next to me, when we arrived at the camp they check your things, and she had condoms, and [the police] took the condoms away.”* Carolina, 29

#### Pregnancy experience and needs during transit

Both first- and second-hand accounts of pregnancy in the Darién Gap were recorded. Most interviewees said that they saw at least one visibly pregnant woman along the journey, with one participant mentioning that pregnant women travel through the Darién Gap *“in very large quantities”* (Jesus, 37). Similar to other health service provision within the Gap, there were *“no doctors helping [pregnant] women [along the way]”* (Osei, 32). Pregnant women must bring their own medicines or supplements with them on the journey, and they often get lost, damaged or stolen along with other possessions. While the three female participants who were pregnant at the time of interview seemed to be fully conscious of the risks, they expressed feelings of acute anxiety, guilt, and the overwhelming weight of maternal responsibility.

Gabriel, 26, recounted witnessing a migrant woman give birth in the forest who received emergency help:

*“Gabriel: There was a girl who gave birth there, gave birth […] a truck arrived and took her*.

*Interviewer: Took her where, sorry?*

*Gabriel: They took her to the hospital, to the labour ward, she had pains in the forest.”*

However, Beatriz's story of seeing a pregnant woman who also started giving birth along the way showed a different angle:

*“She was pregnant, her water broke, you could see from her belly. She lifted her robe […] and you could see the baby moving […] Having a paramedic, someone to help her there in the jungle, there, maybe that could have saved the baby, I don't know if they saved it or not.”* Beatriz, 40

This disparity may point to a potential inconsistency in emergency service response, alongside the absence of regular health service provision in the Gap. This was highlighted by Carolina's care experience at the Darién MRS:

*“I felt that I had [the foetus] very low, I couldn't close my legs and I was very worried. I talked to the people [at the Darién MRS] and there was no medical attention until 3 in the afternoon […] I told them that I felt very poor, and they asked me “Are you bleeding?” I told him “No”. He responded that [the problem] doesn't count as essential.” Carolina, 29*

The other two pregnant participants, Ana and Sonia, gave a somewhat positive account of their experience at the MRS—both women said they were given vitamins and folic acid at the MRS and were overall satisfied with the services. Ana mentioned that she *“wouldn't have received the same support, nor the same attention”* in Panama if it wasn't for her pregnancy.

#### “Love in the mud”—sex, relationships, and transactional sex

Many participants, such as Mauricio and Jesus, believed that migrant people who have sex on their journey through the Gap have *“dirty mentalities”* and are *“unhygienic”,* saying that *“in all those places it smells of everything except love”.* Sex along the way was viewed by most as *“energy that is wasted”* (Carolina, 29) and that, even with existing partners, there is often *“no intimacy”* (Jaime, 29). However, many have also told stories of seeing couples get together in the Darién Gap:

*“There is something called ‘love in the mud’ here […] There are people who meet on this journey and get into relationships. They become a couple! Man with man or woman with woman, and vice versa […] They meet and suddenly they are together. I met a couple and I thought they’ve been together for ages, turns out that they met about 5 days ago […] I was shocked, and they told me “No, this is ‘mud love’”*. Jesus, 37

One of the participants recounted his own *“one-night stand”*, which happened just before entering the forest in Colombia:

*“It [one-night stand] was there, in Necocli, where I spent 4 days camping on the beach and it was good, that's where the opportunity arose […] I only met her in those 4 days that I was there, the last two days. The girl's name was [name redacted], a very pretty little thing, she was also going, she was going there [through the Gap].”* Simon, 29

Such wide range of attitudes surrounding sexual relationships in transit through the Darién Gap suggests that individual experiences of sex and relationships, as well as the reasons for engaging in them, can be profoundly heterogenous. While the brutal conditions of the journey can have a significant effect on mental and physical health, it would be misguided to describe all sexual and romantic relationships that are formed on the way as wholly negative. The patterns of sexual behaviour during migration, although often significantly influenced by migration itself, appeared to reflect participant's pre-existing views on sexual norms.

##### Transactional sex

Regarding sexual activity in exchange for money, participants gave direct and indirect witness accounts of migrant women providing sexual services in the Gap for food or cash to continue their journey:

*“The men offer the money. And since the girl doesn't have money to keep going and they have children, then they sacrifice themselves for the children, […] because they need the money to, to continue the journey.”* Jaime, 29

Some said that the financial power imbalance between migrant women travelling through the Gap and the men who are soliciting sex means that *“the situation lends itself”* to the exchange (Gabriel, 26).

Second-hand accounts of transactional sex within the Gap were not described by participants as sexually exploitative or non-consensual. Both male and female participants acknowledged that transactional sex is often a commonplace solution to a dire economic situation, and the need for HIV/STI prevention and testing provisions for female sex workers was highlighted:

*“They [those exchanging sex for something] also have to take care of themselves, if that is their job, if that is what they are going to live on. They have to take pills, take good care of themselves.”* Isabel, 18

#### Vulnerability to exploitation and sexual violence

Constant fear of sexual violence could be traced across most of the interviews with female participants. There was a shared agreement that travelling as a woman comes with perceivable risks, even where it wasn't spoken about directly. Many participants gave accounts of the *“organised armed groups”* (Jorge, 28) and *“bandits”* (Mauricio, 26) within the Darién Gap picking out women from travel groups to rape during or after the robbery.

Participants described how during the incident they were *“put together”* (Teresa, 20) as a group by the armed men and robbed, with some participants also mentioning that they *“could see what they [the armed men] were doing”:*

*“They started choosing the girls. They chose two young girls whom they’ve gone to rape. They came again for the two more. They raped them again. When I asked what happened before we came, [a friend] said they raped other girls before that. We couldn't see them, because they told us to turn our heads, to lie flat on our stomachs. There, they raped little girls in front of their dad, in front of their parents.”* Francisco, 26

When discussing the dangers encountered on the journey, Mauricio mentioned that he chose to travel on his own instead of bringing his female partner—*“I don't want to travel here with her, because she's a woman”*. This may suggest that even though there is a shared sentiment among the participants of not knowing *“what I was getting myself into”* (Diana, 22), the ubiquity of sexual violence in the Gap is largely recognised.

Participants who discussed incidents of sexual assault in the Gap also discussed the lack of support from the local authorities in regards to reporting cases of sexual violence. Crimes committed on Panamanian territory are the responsibility of the Public Ministry, to whom SENAFRONT typically report all cases of rape. Some participants, like Alvaro, said they felt that the opportunities to file a sexual assault report were limited:

*Interviewer: “They reported that they were raped to the police, or did you report it?”*

*Alvaro: “Yes, but they don’t say anything. We told the soldiers, but they didn’t tell us anything. They said “Yes, we are after them, but what can we do? The jungle is huge, so what can we do?”*.

The feelings of shame, guilt, and fear were mentioned as the primary reasons for not reporting cases of rape, suggesting that women did not report *“out of fear”* and *“because of what happened to them”.* Alvaro said that women who were raped within the forest *“mostly ke[pt] it to themselves, they [were] ashamed to talk about it”.* He mentioned that when a woman in his travel group was raped *“the husband told her not to speak [about it]”.* In one case, participant Teresa talked about a woman whom she witnessed was *“reporting [the rape]”* at the Darién MRS, *“a girl who was bleeding”*, and that *“[local authorities] took her to the doctors”*.

Some participants expressed the view that emergency contraception should be provided along the journey to mitigate the unintended consequences of rape:

*“[Armed men] are raping [the women], imagine that they get pregnant, it's not their fault,”* said Mauricio. Maria said that a young woman travelling in her group *“told her mom to stay calm [about the rape], as long as she doesn't get pregnant”*. Similar to regular contraception, participants considered it necessary *“to bring stuff [emergency contraception] with you”* (Isabel, 18), although did not know how or where the contraception was acquired. This may point to the fact that women who were preparing to travel through the Darién may have been aware of the likelihood of sexual violence and so had planned to acquire the emergency contraception before the start of the journey.

## Discussion

Migrants transiting through the Darién Gap between Colombia and Panama gave accounts of their gruelling journey, which often manifested in fear, anxiety, anger, and the feeling of being at the mercy of not only the difficult conditions of the terrain but also of the individuals involved in the migratory process. Many expressed an opinion that every part of the journey, from being taken through the forest by “coyote” guides, to being left near the Costa Rican border by the National Migration Service, was an inter-connected *“business”* between both lone and state actors. However, whilst participants knew that they were a commodity to human traffickers, they believed that travelling through the Darién was their only option to reach North America.

The resultant desperation of those wishing to emigrate can render them particularly vulnerable, making them an easy target for exploitation and violence. A sense of helplessness and vulnerability was intensified by the perceived ubiquity of sexual violence and a lack of a functional reporting system concerning sexual assault within the Gap. Among a sample of 52 men and 45 women interviewed by Gabster et al. as part of their rapid epidemiological SRH assessment, 40.4% (*n* = 21) of men and 54.8% (*n* = 23) of women felt somewhat or completely unsafe from sexual assault while traveling through the Gap (8). Garbett et al.'s systematic review of Central American data on SRHR challenges of migrant women and girls further supports the findings of our study—the absence of a coordinated multisectoral approach to the SRHR of migrant people, as well as a lack of consideration of issues surrounding their proactive agency and autonomy, negatively affect the wellbeing and health outcomes of migrant women, which can increase the incidence of GBV (26). The absence of wider social protection policies and international guidelines regarding the health and protection of migrant people often make it difficult for women to be active agents in their SRHR, instead placing an emphasis on disease transmission and perceived promiscuity. This can reinforce existing stigma regarding both migrant status and sex work or transactional sex (26, 27).

According to PAHO, migrant people are reportedly less likely to seek help from the national health systems during transit for the treatment of infectious diseases, including HIV, tuberculosis, viral hepatitis, and STIs (28). Kentikelenis et al. drew on the evidence from European economic crises to argue that migrant people can be at a disproportionately higher risk of STIs specifically related to unemployment and poverty, which create the conditions for HIV/STI transmission due to increased exposure to other risk factors. For example, changes in one's socioeconomic environment can affect risk factors for intravenous drug use and transactional sex (29). Following the concept of “paradox of agency”, originally put forward by Garbett et al.,in a context of constrained choices, many of the SRHR challenges migrant women and girls face can act as both a risk and a coping mechanism. For example, a decision to engage in transactional sex during the journey, may have short-term benefits of advancing on the migration journey by ensuring a flow of income, while simultaneously increasing longer-term health risks and making the person more vulnerable to violence. Using a contemporary paradigm supported by the Women's Refugee Commission, those engaging in transactional sex should not be characterised by victim/saviour relationships or be viewed as rescue cases (30, 31).

In our study, participants reported poor gynaecological care for women with urinary, reproductive tract, and sexually transmitted infections, a lack of preventative SRH care, unhygienic communal bathrooms, and an absence of dedicated services for pregnant women were perceived by the participants as factors that didn't allow for adequate personal care for those passing through the Darién MRS. Clinical screening data collected by Toller-Erausquin *et al*. in early 2022 revealed that of a sample of migrant men and women screened, 58.8% (*n* = 30/51) of women and 5.7% (*n* = 4/70) of men reported a current reproductive tract or genital infection symptom, with abnormal vaginal discharge being the most common symptom among the women screened (40.0%) (8). Furthermore, SENAFRONT appeared to confiscate condoms upon arrival to the MRS, leaving people with a choice to either abstain from sexual activity or to engage in riskier sexual relationships.

Sexual behaviours are often significantly influenced by the process of migration itself, as migrant people extricate themselves from the social circles in their own countries, becoming part of different, often larger sexual networks (32, 33). Quantitative studies among migrant people in South Africa, Portugal, and Belgium have demonstrated that “risky” sexual behaviour and HIV acquisition were more prevalent among male and female migrant people compared to their local counterparts, both in transit and after re-settlement (34–39). Toller-Erausquin et al. data showed that out of a sample of migrant men and women who had a casual sexual partner in the last month, 42.9% (*n* = 9/21) of men and 80.0% (*n* = 8/10) of women reported not using a condom; whilst 32.7% (*n* = 18/55) of men and 18.2% (*n* = 8/44) of women reported never having an HIV test prior to the study (8). Lack of HIV/STI prevention methods (ie. condoms) and an absence of corresponding oral communication from the health staff contributes to an inability of making informed personal choices rooted in one's bodily autonomy and agency. This is another instance of the potential “paradox of choice” among migrant people in transit—for a migrant woman, having short-term sexual relationships may deter sexual advances from other men and provide a feeling of safety, but in an environment where it is logistically difficult to practice safer sex, both options carry risks (26).

Tied in with reduced access to HIV/STI prevention methods, pregnancy care, and help after sexual assault and rape were conspicuously absent in this setting, which does not align with established humanitarian standards, such as the Minimum Initial Service Package (MISP) for Sexual and Reproductive Health in Crises, supported by the Woman Refugee Commission under the UNHCR (40, 41). MISP standards are to be applied to all people affected by humanitarian emergencies, including migrant people in vulnerable situations, as all individuals “have a fundamental human right to reproductive healthcare” (41). Furthermore, MISP services can be implemented without an in-depth needs assessment due to the amount of existing evidence that justifies its use, so there is currently no reason for why migrant people in transit in Panama (and elsewhere) can be denied such care. The right to reproductive care cannot be exercised without the availability of MISP services, including the management of consequences of sexual violence, prevention of excess maternal and new-born morbidity and mortality, and provision of contraceptives.

Based on the WHO data, approximately 15% of women pregnant at the time of displacement will experience obstetric complications, including “obstructed or prolonged labour, pre-eclampsia/eclampsia, sepsis, ectopic pregnancy or complications of abortion”, many of which will not manifest in external bleeding (41, 42). Obstetric care that is not consistently available 24 h per day in humanitarian settings is shown to have a significant effect on increasing maternal and neonatal morbidity and mortality (43). There is currently no evidence of MISP provision targeted at migrant people in transit at Darién MRS or elsewhere in Panama, outside of the general health assistance provided by MSF and the Panamanian Red Cross. Furthermore, research done by Holloway et al. (44) of the Humanitarian Policy Group reiterated that SRHR of migrant peoples were not properly addressed in other Latin American countries along the route to Panama. For example, in Colombia, despite MISP implementation by PAHO/WHO, the local coordination was weak, and SRH services were not adequately integrated with HIV and GBV programmes, leading to vulnerable individuals “likely falling through the cracks” (45).

This study had a number of strengths and limitations. The use of semi-structured interviews as the main instrument for this qualitative study was a suitable approach for giving participants a voice in the collection of their experiences, feelings, and opinions. The approach enabled a more relaxed, trusting interview environment, and helped achieve data saturation. This study had three main limitations. First, an overwhelming majority (*n* = 20/26) of interviewees originated from Venezuela, which limited the diversity of participant background. During participant recruitment, every effort was made to diversify the participant sample to reflect the range of personal experiences, views, and beliefs; however, the selection process was based on ascertaining the most information-rich cases and the suitability of participants based on the pre-determined inclusion/exclusion criteria– at the time of data collection, limited number of migrant people in transit from countries outside of Venezuela were deemed to fit the above conditions. Six participants who originated from countries outside of Latin America were also all male, which may have further affected representativity of the sample, however the fact that nearly all individuals originating from extracontinental countries are male; therefore, this is in line with what Toller-Erausquin et al’s article also found. Nevertheless, participant interviews provided a sufficient range of information to reach data saturation regarding all four a-priori defined themes. Second, the externality of one of the interviewers being a white, English-speaking woman may have introduced respondent bias into participants' responses, but was minimised by employment of well-trained, Spanish-speaking local staff where required. Thirdly, given the small participant sample of our study, the findings cannot be generalisable to all migrants in transit through Panama or Latin America; further longitudinal research across several countries is required in the future to help build a picture of SRH services needs and outcomes associated with the migrant people's journey. Fourthly, non-response bias could have occurred; those who were at greatest risk or those who were survivors of assault may not have accepted to participate in the study.

In conclusion, this study has captured crucial insights into the experience and unmet needs regarding SRH care and access to services within the Darién Gap, at Darién reception centre, and while travelling across Panama. Lack of appropriate risk-prevention information, reduced access to contraception, HIV/STI testing, and antenatal care pertains to the erosion of bodily autonomy and changes in sexual behaviour, both of which are intensified by the gruelling migration process. Local SRH interventions at the Panamanian reception centres and mobilisation of personnel are required to mitigate adverse health outcomes for migrant people in transit through the country. Many survivors are reluctant to report sexual and other GBV to authorities due to the bureaucracy of referral process. Therefore, integration of SRH services with GBV programmes is key—safe and confidential spaces within Panamanian MRS should be available to provide survivors with appropriate clinical care, and fast-tracked health referrals. On a larger scale, improved cooperation between governmental and non-governmental agencies, and provision of a minimal package of SRH preventative and care services throughout the migration route, are required to address the needs of highly vulnerable migrant populations.

## Data Availability

The raw data supporting the conclusions of this article will be made available by the authors, without undue reservation.
